# *GeneXpert MTB/RIF* dans le dépistage de la tuberculose pulmonaire à l’Hôpital Provincial Général de Référence de Bukavu, à l’Est de la République Démocratique du Congo: quelles leçons tirées après 10 mois d’utilisation?

**DOI:** 10.11604/pamj.2017.27.260.12575

**Published:** 2017-08-08

**Authors:** David Lupande, David Kaishusha, Carine Mihigo, Moise Itongwa, Gustave Yenga, Philippe Katchunga

**Affiliations:** 1 Département de Biologie Médicale, Service de Microbiologie/Hôpital Provincial Général de Référence de Bukavu, Université Catholique de Bukavu, B.P 285, Bukavu, RD Congo; 2 Département de Biologie Médicale, Service de Microbiologie/Cliniques Universitaires de Kinshasa, Université de Kinshasa, B.P 834 KIN XI, RD Congo; 3 Institut Supérieur de Techniques Médicales de Nyangezi, Sud-Kivu, RD Congo; 4 Institut Supérieur de Techniques Médicales de Nyangezi, Sud-Kivu, RD Congo

**Keywords:** GeneXpert MTB / RIF, tuberculose, dépistage, Bukavu, GeneXpert MTB / RIF, tuberculosis, screening, Bukavu

## Abstract

**Introduction:**

En Afrique subsaharienne, les méthodes de diagnostic de la tuberculose sont insuffisantes et reposent essentiellement sur la microscopie. Elles constituent un réel frein pour le contrôle de la tuberculose. La présente étude voudrait évaluer les performances du *GeneXpert MTB/RIF* vis à vis de la microscopie classique de Ziehl-Neelsen à l’Hôpital Provincial Général de Référence de Bukavu, à l’Est de la République Démocratique du Congo après 10 mois d’utilisation.

**Méthodes:**

Les résultats de la coloration au Ziehl-Neelsen et de la biologie moléculaire sur *GeneXpert MTB/RIF* de 452 patients suspects de tuberculose ont été colligés. La validité d’un test par rapport à l’autre dans la détection de la tuberculose a été étudiée.

**Résultats:**

Dans le groupe entier, la fréquence de la tuberculose pulmonaire était de 16.3%. La positivité était significativement plus élevée pour le GeneXpert MTB/RIF que pour le Ziehl-Neelsen dans le groupe entier (15.9% vs 9.3%, p= 0.03) et chez les séropositifs pour le VIH (52.0% vs 24.0%; p = 0.007). Cependant, la sensibilité de *GeneXpert MTB/RIF* comparé au Ziehl-Neelsen n’était pas maximale (95.2%). Enfin, *GeneXpert MTB/RIF* a détecté 20.8% de résistance à la rifampicine.

**Conclusion:**

La présente étude confirme la supériorité de *GeneXpert MTB/RIF* sur la coloration de Ziehl-Neelsen dans la détection de la tuberculose et dans la prédiction de la multi résistance. Son utilisation systématique couplée au Ziehl-Neelsen permettrait de mieux contrôler la tuberculose en Afrique subSaharienne.

## Abstract (en)

**Introduction:**

In sub-Saharan Africa, diagnostic methods for tuberculosis are inadequate and are essentially based on microscopy. They constitute a real obstacle to the control of tuberculosis. This study aimed to evaluate the performance of GeneXpert MTB/RIF test compared to classical Ziehl-Neelsen staining at the the general referral provincial hospital of Bukavu, in the east of the Democratic Republic of the Congo after 10 months of use.

**Methods:**

The results of Ziehl-Neelsen staining and GeneXpert MTB/RIF molecular biology test performed in 452 patients with suspected tuberculosis were collected. This study compares the validity of these different diagnostic tests in the detection of tuberculosis.

**Results:**

In the entire group, the frequency of the pulmonary tuberculosis was 16.3%. The positivity rate was significantly higher in GeneXpert MTB/RIF test than in Ziehl-Neelsen staining in the entire group (15.9% vs 9.3%, p = 0.03) and in HIV seropositive patients (52.0% vs 24.0%; p = 0.007). However, the sensitivity of GeneXpert MTB/RIF test compared to that in Ziehl-Neelsen staining wasn’t maximum (95.2%). Finally, GeneXpert MTB/RIF test detected rifampicin resistance in 20.8%.

**Conclusion:**

This study confirms the superiority of GeneXpert MTB/RIF test compared to Ziehl-Neelsen staining in the detection of tuberculosis and in the prediction of multi-resistance. Its systematic use coupled with Ziehl-Neelsen staining would better control tuberculosis in sub-Saharan Africa.

## Introduction

La Tuberculose (TBC) est un sérieux problème de santé publique mondial. En effet, les statistiques de l’Organisation Mondiale de la Santé (OMS) notaient 9 millions de nouveaux cas de TBC et 1.5 millions de décès dont 360.000 séropositives pour le virus de l’immunodéficience humaine (VIH) en 2013 [1]. En outre, environ 480.000 nouveaux cas de TBC multi-résistante ont été diagnostiqués dans le monde, soit 3,5% de tous les cas de TBC au cours de la même année et, seules 97.000 ont bénéficié d’un traitement efficace parmi lesquelles seulement 48% ont été guéris [1]. La morbidité et la mortalité liées à la TBC sont très élevées dans les pays à revenu faible et intermédiaire. Singulièrement, l’Afrique subSaharienne (ASS) enregistre généralement la plus grande proportion de nouveaux cas par habitant avec 280 cas pour 100.000 habitants [1]. De même, la mortalité due à la tuberculose de 44% en ASS reste très élevée [1]. Et c’est toujours dans ce continent que la prévalence de l’infection à VIH demeure très élevée [2] expliquant également une fréquence élevée de la co-infection VIH-TBC [1, 3, 4]. Malheureusement, c’est dans ce continent que les méthodes de diagnostic de la TBC restent insuffisantes reposant essentiellement sur la microscopie au niveau des structures de santé de premier niveau, la culture se faisant uniquement dans certaines structures de santé du niveau tertiaire. La sensibilité de la microscopie dans la détection de nouveaux cas et la co-infection VIH-TBC est faible et devient ainsi un réel frein pour le contrôle de la TBC [1]. Des méthodes de diagnostic basées sur la biologie moléculaire sont actuellement proposées, dont deux ont été validées dans différents programmes de pays en voie de développement par l’OMS, le *GenoType MTBDR* plus assay (Hain Lifescience GmbH, Nehren, Germany) et le *Xpert MTB/RIF* assay (Cepheid, USA) [5, 6]. Ces méthodes de dépistage sont en principe extrêmement sensibles, spécifiques et rapides. Cependant, la limite de la PCR est son coût excessif. En République Démocratique du Congo (RDC), le Programme de lutte contre la Tuberculose (PNLT) a toujours été parmi les mieux organisés du pays. Malheureusement, parmi les 22 pays portant la charge de la Tuberculose à 80% dans le monde, la RDC occupe la 11^ème^ position, avec une incidence estimée à 549 pour 100000 habitants par an [1, 7]. Ainsi, pour augmenter la détection de cas de TBC, le PNLT a doté les principales structures de santé du pays en automate *GeneXpert MTB/RIF* dont l’Hôpital Provincial Général de Référence de Bukavu (HPGRB), structure de référence de la province du Sud-Kivu, à l’Est de la RDC. Dans cette institution de santé, la TBC constitue 3,3% des admissions globales en médecine interne et 12,6% des admissions pour maladies transmissibles (registres de statistiques). Ainsi, la présente étude voudrait évaluer les performances du *GeneXpert MTB/RIF*, vis à vis de la microscopie classique de Ziehl-Neelsen (ZN) ainsi que la prévalence de la résistance à la rifampicine déterminée par l’automate à l’HPGRB.

## Méthodes

### Type d’étude

La présente étude rétrospective, descriptive et analytique a consisté en une collecte, à partir des registres de l’unité de Bactériologie de l’HPGRB, des données démographiques (sexe, âge), des résultats simultanés de ZN et du GeneXpert MTB/RIF ainsi que de la sérologie anti VIH de 452 patients suspects de tuberculose pulmonaire durant la période allant du 1er février 2012 au 31 décembre 2012. Ces patient(e)s étaient d’emblée examinés par un interniste ou généraliste et des examens d’orientation (hémogramme, biologie inflammatoire, radiographie du thorax) étaient réalisés. Toutes les règles de confidentialité et d’éthiques telles que prescrites dans la déclaration d’Helsinki ont été respectées. Avant la récolte de données, les autorisations respectives du Médecin Directeur de l’HPGRB et du Médecin coordonnateur provincial du Programme National de Lutte contre la Tuberculose avaient été obtenues. Les données étaient collectées de manière anonyme.

### Définitions opérationnelles

Etait tuberculeux dans la présente étude tout patient avec ZN et/ou *GeneXpert MTB/RIF*. Etait résistant à la rifampicine tout patient dont la résistance à la rifampicine était confirmée par le *GeneXpert MTB/RIF*.

### Analyses statistiques

Les données de la présente étude sont présentées sous des moyennes ± déviation standard ou de fréquences selon les cas. Pour la comparaison des variables, le test « chi carré » de Pearson a été utilisé pour les proportions. L’étude de la validité du test de ZN comparé au *GeneXpert MTB/RIF* et vice versa a été réalisée. Pour ce faire, nous avons utilisé le logiciel MedCalc^®^ 12.4.0 software. Une valeur de p < 0,05 était considérée comme statistiquement significative.

## Résultats

### Caractéristiques générales de la population étudiée

Le Tableau 1 reprend les caractéristiques générales de la population étudiée. Au total, sur les quatre cents cinquante-deux (452) patients suspects de Tuberculose pulmonaire ayant réalisé simultanément la coloration de ZN et la PCR avec le *GeneXpert MTB / RIF*, 224 (49.6%) étaient des hommes et 228 (50.4%) des femmes. La moyenne de l’âge de l’ensemble du groupe était de 37.0 ± 15.7 ans.

**Tableau 1:** Caractéristiques générales de la population étudiée

	**n (%)**
Total	452 (100)
Hommes	224 (49.6%)
Femmes	228 (50.4%)
Age (ans)	37.0±15.7
**Tranche d’âge (ans)**	
<20	37 (8.2%)
20-39	259 (57.3%)
40-59	105 (23.2%)
≥60	51 (11.3%)

Fréquence de la tuberculose pulmonaire

La fréquence de la tuberculose est mentionnée dans le Tableau 2. De ces 452 patients suspects, la tuberculose pulmonaire a été confirmée chez 74 patients soit une fréquence de 16.4%. Elle concernait 45 (20 %) des hommes contre 29 (12.6 %) femmes, soit un sex ratio de 1.55 H/1 F, la différence étant statistiquement significative (p = 0,04). Comparé au ZN, la positivité avec le *GeneXpert MTB/RIF* était significativement plus élevée (15.9% vs 9.3% ; p = 0,003). De plus, parmi 50 patients séropositifs pour le VIH, la positivité avec le *GeneXpert MTB / RIF* était plus élevée qu’avec le ZN (52.0% vs 24.0%; p = 0,007).

**Tableau 2:** Fréquence de la tuberculose pulmonaire

	**n (%)**
**Sexe**	
Hommes et femmes	74 (16.4)
Hommes	45 (20.0)
Femmes	29 (12.7)
**p**	0.04
**Diagnostic biologique**	
PCR positive	72 (15.9)
Ziehl-Neelsen positif	42 (9.3)
**P**	0.003
**Patients VIH positifs (n=50)**	
PCR positive	26 (52.0)
Ziehl-Neelsen positif	12 (24.0)
**p**	0.007

Validité de GeneXpert MTB/RIF et de Ziehl-Neelsen

Les Tableau 3 et Tableau 4 montrent la validité respectivement du Ziehl-Neelsen comparé au *GeneXpert MTB / RIF* et de *GeneXpert MTB/RIF* comparé au Ziehl-Neelsen. Il en ressort que parmi les 72 patients positifs au GeneXpert MTB/RIF, seuls 40 (55.6%) patients avaient un Ziehl-Neelsen positif versus 32(44 .4%) qui étaient négatifs au Ziehl-Neelsen (Tableau 3). Et parmi 380 patients négatifs au GeneXpert MTB/RIF, 378 (99.5%) étaient également négatifs au Ziehl-Neelsen (Tableau 3). Parmi 42 patients positifs au Ziehl-Neelsen, 40 (95.2%) étaient également positifs au *GeneXpert MTB / RIF* contre 2(4.8%) qui étaient négatifs au *GeneXpert MTB / RIF* (Tableau 4).

**Tableau 3:** Validité du test de Ziehl-Neelsen comparé au *GeneXpert MTB/RIF*

	PCR GeneExpert MTB/RIF	
	**Positif** **n= 72 (15.9%)**	**Négatif** **n= 380 (84.1%)**
**Ziehl-Neelsen**		
Positif	40 (55.6%)	2 (0.5%)
Négatif	32 (44.4%)	378 (99.5%)

**Tableau 4:** Validité du test de *GeneXpert MTB/RIF* comparé au Ziehl-Neelsen

	**Ziehl-Neelsen**	
	**Positif** **n= 42 (9.3%)**	**Négatif** **n= 410 (90.7%)**
**PCR *GeneXpert MTB/RIF***		
Positif	40 (95.2%)	32 (7.8%)
Négatif	2 (4.8%)	378 (92.2%)

### Fréquence de la résistance à la rifampicine

Parmi les 72 patients positifs au *GeneXpert MTB / RIF*, 15 (20.8%) avaient une résistance à la rifampicine.

## Discussion

Le présent travail a noté une fréquence de la tuberculose pulmonaire de 16.3% parmi 452 patients cliniquement suspects de cette maladie. La positivité était significativement plus élevée pour le *GeneXpert MTB / RIF* que pour la coloration de ZN spécifiquement chez les séropositifs pour le VIH. Comparé au test de *GeneXpert MTB / RIF*, le ZN avait une sensibilité faible (55.6%). Néanmoins, comparé au ZN, la sensibilité de *GeneXpert MTB / RIF* F n’était pas maximale (95.2%). Enfin, parmi les 72 patients positifs au GeneXpert MTB/RIF, 15 (20.8%) avaient une résistance à la rifampicine. La fréquence de la tuberculose trouvée dans le présent travail se rapproche de celle rapportée par d’autres auteurs. En effet, Gounder A et al ont noté, sur un échantillon de 415 patients suspects, une fréquence de la tuberculose de 18,7% au Fiji [8]. La fréquence était plus élevée en Turquie (26%) [9]. Par contre, Sekkade et al. en Uganda, un pays à forte prévalence au VIH, ont relevé une fréquence de la tuberculose relativement basse de 14% chez 250 enfants de 2 à 12 ans dont 41,6% étaient séropositifs pour le VIH [10]. Cette variation pourrait s’expliquer par la différence dans la méthodologie appliquée et surtout par les facteurs de risque particulièrement, l’infection à VIH. D’après l’OMS, sur les 9.6 millions de nouveaux cas de tuberculose dans le monde, 1.2 millions étaient séropositives à VIH en 2014, soient 13% [1]. Dans notre série, la prévalence de la coinfection VIH/Tuberculose était relativement plus élevée que celle de l’OMS. Ceci confirme la forte corrélation entre la coinfection VIH/TBC et la prévalence du VIH dans une région. En effet, le VIH multiplie le risque de développer une tuberculose de 24 à 31 fois [1]. La présente étude a également permis de relever la forte validité de *GeneXpert MTB / RIF* dans la détection de la TBC comparé au ZN.

Cependant, la sensibilité de *GeneXpert MTB / RIF* comparé au ZN n’était pas maximale. Nos résultats corroborent ceux d’autres auteurs notamment Geleta et al. en Ethiopie [11], Giang et al. au Vietnam [12] et Lawn et al. en Afrique du Sud [13]. Pour certains auteurs, Le couple ZN / PCR conventionnelle permet de détecter plus de cas de TBC que le ZN seul [14]. Notre étude a aussi montré une sensibilité élevée de GeneXpert MTB/RIF dans la détection de la TBC chez les personnes vivant avec le VIH en particulier corroborant ainsi d’autres études [1, 15]. Enfin, l’utilisation de *GeneXpert MTB / RIF* a permis de détecter une fréquence de 20.8% de Résistance à la Rifampicine, marqueur de la prédiction de la multirésistance pour la tuberculose. Cette fréquence semble significativement plus élevée que celles trouvées dans d’autres études et dépasse largement l’estimation de l’OMS qui est de 10%. Cependant, Steingart et al, sur l’utilisation du *GeneXpert MTB / RIF* et la détection de la résistance à la rifampicine, préconisent une prudence quant à la prévalence de la résistance, dans la mesure où le taux de faux positif et de faux négatifs dépendront de la prévalence de la résistance localement, de l’existence de l’antibiogramme en milieux liquides, de la culture du *Mycobacterium tuberculosis* et de fois jusqu’au séquençage pour confirmer [16]. De même, Sanchez-Padilla et al ont démontré que le *GeneXpert MTB / RIF* ne sait pas détecter toutes les mutations du gène rpoB, et pour ces genres d’isolats, l’automate les classera sensibles [17]. La limite majeure de la présente étude est qu’elle est rétrospective impliquant ainsi toutes les insuffisances inhérentes à cette méthodologie notamment le manque de certaines informations sur le suivi et l’évolution des malades une fois le diagnostic posé, surtout ceux avec résistance à la rifampicine. La culture du Complexe Mycobacterium tuberculosis (Gold standard) n’était pas faisable dans le laboratoire de Microbiologie, ce qui n’a pas permis de l’utiliser comme examen de référence dans la présente étude. En dépit de ces limites, cette étude a eu le mérite d’avoir évalué pour la première fois le *GeneXpert MTB / RIF* implanté à l’Hôpital Provincial à Bukavu durant ses 10 premiers mois d’utilisation.

## Conclusion

La présente étude confirme une validité significativement plus élevée de *GeneXpert MTB / RIF* que de la coloration de ZN dans la détection de la tuberculose et sa place dans la prédiction de la multirésistance. Son utilisation systématique couplée au ZN permettrait de mieux contrôler la TBC dans notre milieu.

### Etat des connaissances actuelles sur le sujet

- Le *GeneXpert MTB/Rif* est un automate qui améliore sensiblement le diagnostic de la tuberculose, surtout chez les personnes à sérologie positive au VIH et chez les enfants qui ne savent pas expectorer, dont seul Ziehl Neelsen ne savait le faire;
- L’épidémiologie de la résistance à la Rifampicine est bien documentée avec le *GeneXpert MTB/Rif*, ce qui nous permet d’anticiper la prise en charge par l’utilisation des molécules anti tuberculeuses plus éfficaces contre les souches multirésistantes.

### Contribution de notre étude à la connaissance

- Notre étude confirme qu'il est nécessaire d'intensifier et de généraliser l’utilisation de l’automate *GeneXpert MTB/Rif* dans le diagnostic et la surveillance de la tuberculose;
- Avec ce travail, il a été clairement démontré qu’à Bukavu, l’usage du *GeneXpert MTB/Rif* a montré les mêmes évidences que celles décrites ailleurs dans le monde, ce qui a poussé l’OMS à l’adopter et à le recommender dans les différents programmes nationaux de pays membres.

## Conflits d’intérêts

Les auteurs ne déclarent aucun conflit d'intérêt.
